# Intestinal IFNα4 promotes 15-HETE diet-induced pulmonary hypertension

**DOI:** 10.1186/s12931-024-03046-z

**Published:** 2024-11-28

**Authors:** Grégoire Ruffenach, Lejla Medzikovic, Laila Aryan, Wasila Sun, Long Lertpanit, Ellen O’Connor, Ateyeh Dehghanitafti, Mohammad Reza Hatamnejad, Min Li, Srinivasa T. Reddy, Mansoureh Eghbali

**Affiliations:** 1 Division of Molecular Medicine, Department of Anesthesiology and Perioperative Medicine, David Geffen School of Medicine, University of California, CHS BH-550 CHS, Los Angeles, CA 90095-7115 USA; 2 Division of Cardiology, Department of Medicine, David Geffen School of Medicine, University of California, Los Angeles, CA 90095-7115 USA

## Abstract

**Objectives:**

Pulmonary arterial hypertension (PAH) is characterized by the remodeling of the pulmonary vascular bed leading to elevation of the pulmonary arterial pressure. Oxidized fatty acids, such as hydroxyeicosatetraenoic acids (HETEs), play a critical role in PAH. We have previously established that dietary supplementation of 15-HETE is sufficient to cause PH in mice, suggesting a role for the gut-lung axis. However, the mechanisms are not known.

**Approach:**

Analysis of RNA-seq data obtained from the lungs and intestines of mice on 15-HETE diet together with transcriptomic data from PAH patient lungs identified IFN inducible protein 44 (IFI44) as the only gene significantly upregulated in mice and humans. We demonstrate that IFI44 is also significantly increased in PBMCs from PAH patients. In mice, 15-HETE diet enhances IFI44 and its inducer IFN⍺4 expression sequentially in the intestine first and then in the lungs. IFI44 expression in PAH is highly correlated with expression of Tumor Necrosis Factor Related Apoptosis Inducing Ligand (TRAIL), which is upregulated in CD8 cells in PH lungs of both mice and humans. We show that IFNα4 produced by intestinal epithelial cells facilitates IFI44 expression in CD8 cells. Finally, we demonstrate that IFN receptor 1-KO in mice do not develop PH on 15-HETE diet. In addition, silencing IFI44 expression in the lungs of mice on 15-HETE diet prevents the development of PH and is associated with significantly lower expression of IFI44 and TRAIL in CD8 cells in the lungs.

**Conclusion:**

Our data reveal a novel gut-lung axis driven by 15-HETE in PH.

**Supplementary Information:**

The online version contains supplementary material available at 10.1186/s12931-024-03046-z.

## Introduction

Pulmonary arterial hypertension (PAH) is a fatal condition in which loss and obstructive remodeling of the pulmonary vascular bed is responsible for the rise of pulmonary arterial pressure [1]. Over time, this increased pressure leads to right ventricular failure and ultimately death [2, 3]. Current treatments have significantly expanded the survival of PAH patients with a 3-year survival rate of 79% [4]. Despite this progress, current care only curbs disease progression, with patients ultimately needing transplantation. As such, there is a dire need for new insights into the physiopathology of PAH to discover new therapeutic avenues.

We and others have shown that the plasma concentration of oxidized fatty acids, such as hydroxyeicosatetraenoic acids (HETEs) [5] are increased in PAH and play a critical role in the pathogenesis of PAH [6–8]. Furthermore, we have previously established that the dietary supplementation of a single oxidized fatty acid, 15-HETE, is sufficient to cause PH in wild-type mice as characterized by increased right ventricular systolic pressure and increased thickness of the pulmonary arteriole vascular wall. [6–8]. We found that T cell-dependent endothelial cell apoptosis was one of the mechanisms underlying 15-HETE-induced PH [6].

Dietary intake participates in health and disease development [9, 10], and there is significant evidence of the involvement of the digestive tract in the development of related cardiovascular [10–14] and pulmonary diseases [15–17]. 15-HETE-fed mice have an increased concentration of not only 15-HETE but also other HETEs in the intestinal epithelial cells. However, the exact role of the gut-lung axis in the development of PH induced by 15-HETE remains elusive.

To unravel the gut involvement in 15-HETE-induced PH, we performed RNA-seq on the lungs and intestines of mice fed a 15-HETE diet and integrated our RNA-seq data with an online available microarray of human PAH lungs [18–20]. Our analysis revealed IFI44 (IFN inducible protein 44) as the only significantly upregulated gene in the small intestine and the lungs of mice on a 15-HETE diet and in the lungs of PAH patients. Furthermore, we demonstrate that CD8 cells upregulate IFI44 and its target TRAIL and promote PH by decreasing the pulmonary vascular bed density. Finally, we demonstrate that inhibition of IFI44 in the lungs of 15-HETE diet mice prevents the loss of pulmonary vascular bed density and the development of PH.

## Results

### Large-scale transcriptomic data reveals IFI44 as a common dysregulated gene between intestine and lungs of PH mice, and human PAH lungs

**Fig. 1:**
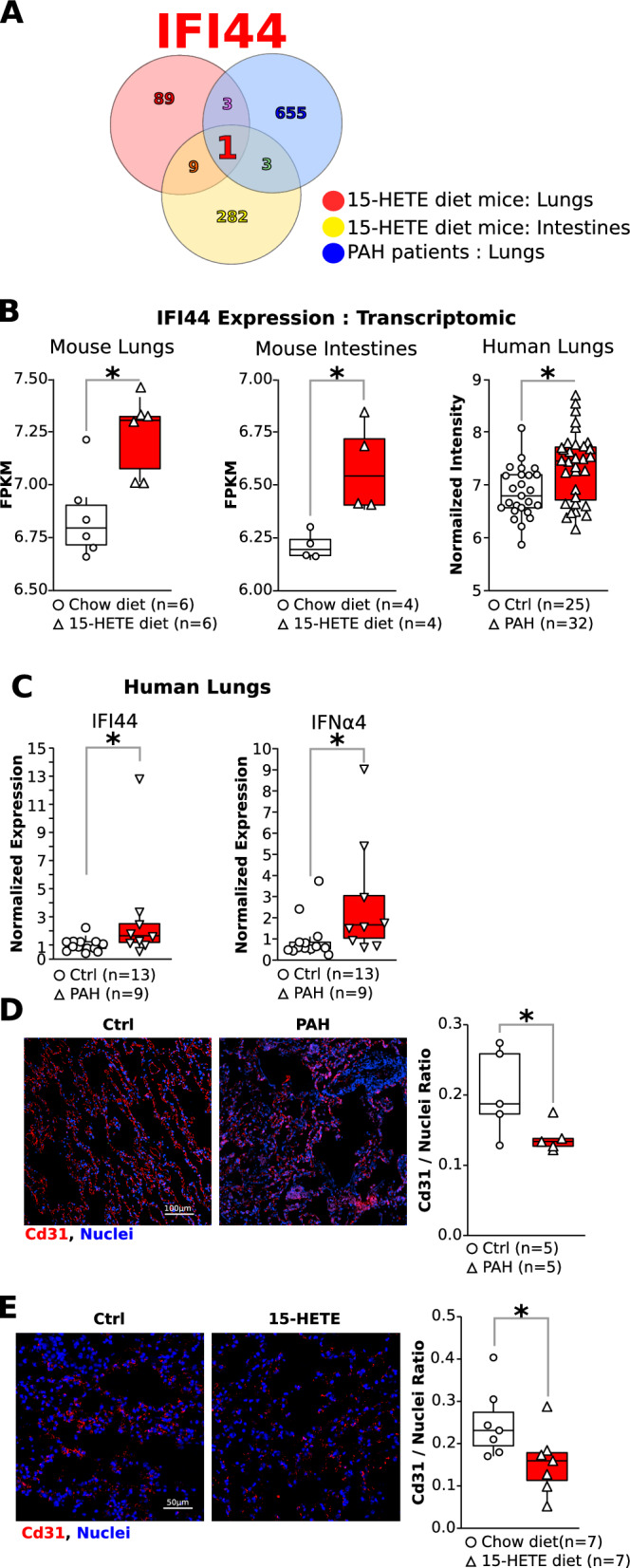
**A** Venn diagram showing the number of genes overlapping between 15-HETE diet mouse lungs and intestines, and PAH patient lungs. **B** Quantification of IFI44 expression in mice lungs and intestines, and human lungs by RNA-sequencing and microarray respectively. **C** Quantification of IFI44 and IFNα4 in human lungs by RT-qPCR in an independent cohort of PAH patients. Representative images and quantification of pulmonary endothelial cell density in **D** humans and **E** in mice. Of Note that some samples in the measurement of IFNα4 and IFI44 are statistical outliers. But without these statistical outliers the up-regulation of IFNa4 and IFI44 remain significant (Suppl Fig 3). We choose to keep these samples as they represent the expression of IFNα4 and IFI44 in PAH patients.

To gain further insight into the role of intestine in PH induced by 15-HETE diet and its relation to the human disease, we performed RNA-seq on intestines of PH mice. We combined the results with our previous RNA-seq on lungs of PH mice on 15-HETE diet [6] and an online-available human PAH lung microarray (GSE117261). This analysis revealed one common differentially expressed gene in all three tissues: IFI44 (interferon-induced protein 44, Fig. 1A). IFI44 was significantly upregulated in the small intestine and lungs of 15-HETE diet-fed mice as well as in human PAH lungs (Fig. 1B). For human, we also confirmed the up-regulation of IFI44 in two other datasets, GSE117261 (25 Ctrl vs 32 PAH), and GSE48149 (9 Ctrl vs 8 PAH) (Suppl. Figure 1). IFI44 was significantly up-regulated in PAH in all three independent cohorts of patients. Of note, IFI44 expression in the lungs of rats with PH induced by MCT or Sugen/Hypoxia was downregulated (Suppl. Fig. 2), in contrast to 15-HETE -induced PH mice and PAH patients, further alluding to the specificity of the 15-HETE diet-induced PH model.

IFI44 is an interferon inducible protein and is known to be up regulated by IFNα4 [26]. We confirmed by qPCR that expression of IFI44 and IFNα4 are significantly upregulated in PAH vs. control lungs in humans (Fig. 1C). We recently showed that mice on 15-HETE diet develop PH and this model recapitulates key hallmarks of PAH such as increased RVSP and vascular remodeling. Here we show that 15-HETE diet mice also recapitulates the decreased pulmonary vascular bed density observed in PAH patients (Fig. 1D & E).

### IFI44 and its target TRAIL are upregulated in CD8 cells in human PAH lungs

**Fig. 2:**
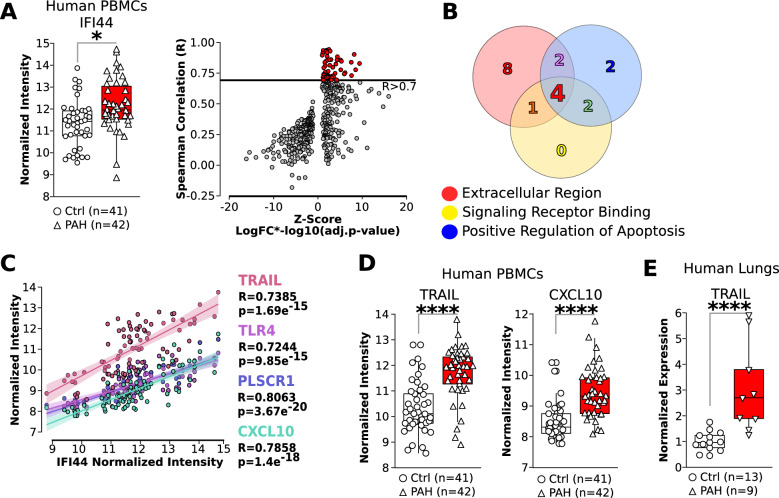
**A** Quantification of IFI44 in human PBMCs measured by microarray and correlation of IFI44 in the same microarray. **B** Venn diagram showing the number of genes that correlate with IFI44 and are annotated for being in the extracellular region, play a role in positive regulation of apoptosis and/or are known to bind to a receptor. **C** Correlation of four genes identified in panel B. **D** Quantification of TRAIL and CXCL10 in human PBMCs by microarray. **E** Quantification of TRAIL in human lungs by RT-qPCR in an independent cohort of PAH patients.

IFNA4 can induce PAH through uncontrolled activation of the immune system [27]. Hence, we focused our attention on the expression of IFI44 by immune cells. In an online-available human microarray from PBMCs from PAH patients [23] we found that IFI44 expression is significantly elevated compared to control samples (Fig. 2A). To investigate the function of IFI44 in immune cells, we correlated the expression of IFI44 with all other dysregulated genes in PMBCs, and found 59 genes to be positively correlated with IFI44 expression (Fig. 2B). We have previously demonstrated that CD8-dependent apoptosis of endothelial cells is one of the mechanisms of PH development in 15-HETE-fed mice [6]. Hence, using gene ontology, we looked for genes that their expression is positively correlated with IFI44 and are *1)* present in the extracellular region, *2)* known to bind to a receptor, and *3)* known to regulate apoptosis. This analysis revealed four genes, TRAIL, CXCL10, TLR4, and PLSCR1 (Fig. 2C). Among these four genes, death receptor ligand TRAIL (Tumor Necrosis Factor Related Apoptosis Inducing Ligand) and proinflammatory cytokine CXCL10 are highly relevant in the context of CD8-dependent endothelial cell apoptosis (Fig. 2D). Since we have recently reported the role of CXCL10 in inducing endothelial cell death in PH [28], here we focused on unraveling the role of TRAIL in regulating EC apoptosis. We confirmed by qPCR that expression of TRAIL is significantly upregulated in the lungs of PAH patients vs. control (Fig. 2E).

### Upregulation of IFI44 in the small intestine precedes its upregulation in the lung of 15-HETE-fed mice

**Fig. 3:**
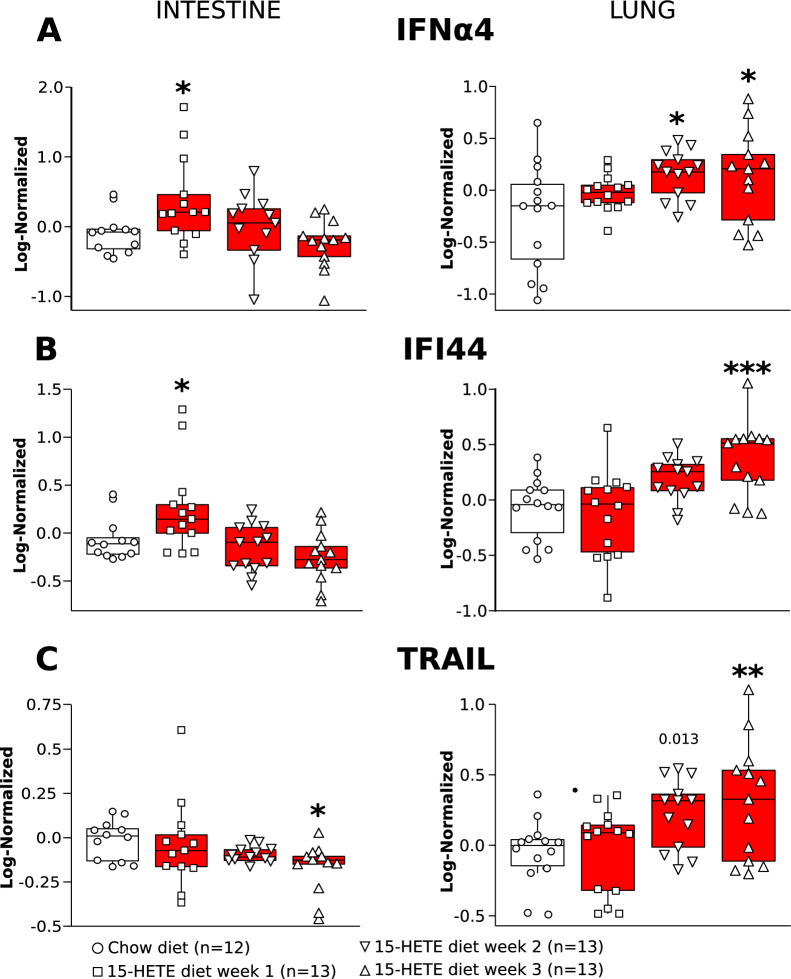
Time course quantification of **A** IFNα4, **B** IFI44, and **C** TRAIL in mice on 15-HETE diet intestines and lungs. *Shows the statistical significance of the mean of a group compared to the chow diet group.

To confirm the involvement of the intestine in PH induced by 15-HETE diet in context of IFI44 activation, we performed a time course experiment to measure IFNα4, IFI44, and TRAIL in the intestine and lungs of 15-HETE diet-fed mice (Fig. 3**)**. This experiment demonstrated that IFNα4 and IFI44 are first upregulated in the intestine as early as 1 week after starting 15-HETE diet and then remain high at week 2 and 3. However, they only started to increase in the lungs during the second week of 15-HETE diet and it is significantly higher at 2 weeks for IFNα4, and at 3 weeks for IFI44, and TRAIL in mice on 15-HETE diet compared to control. These data suggest that 15-HETE may act on the small intestine prior to PH development.

### IFI44 and TRAIL are expressed by CD8 cells in human and mouse lungs

**Fig. 4:**
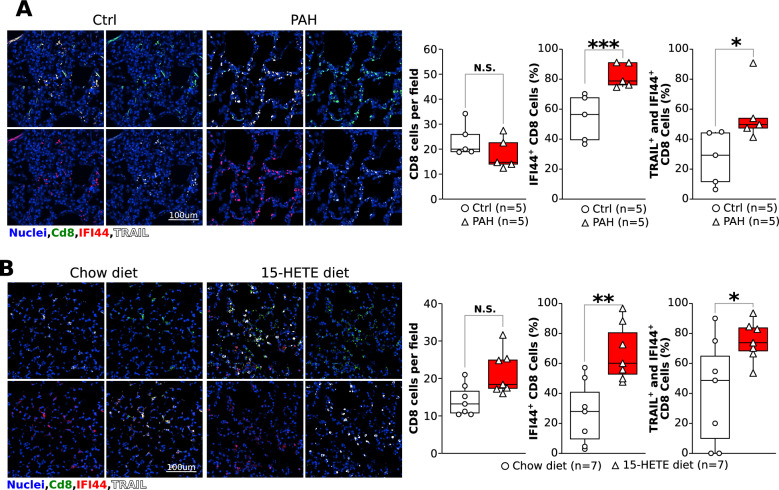
**A** Representative images and quantification of CD8 cells, CD8 cells expressing IFI44, and CD8 cells expressing IFI44 and TRAIL in PAH patients. **B** Representative images and quantification of CD8 cells, CD8 cells expressing IFI44, and CD8 cells expressing IFI44 and TRAIL in mice after three weeks of 15-HETE diet or chow diet.

We have previously shown the role of CD8 cells in 15-HETE induced PH in mice [6], hence we measured the expression of IFI44 and TRAIL in CD8 cells. While there were no significant differences in the number of CD8 cells in lungs of PAH patients and controls, a higher proportion of CD8 cells that express IFI44 or both IFI44 and TRAIL was present in lungs of PAH patients compared to controls (Fig. 4A). This phenotype is recapitulated in the lungs of 15-HETE diet mice, since the expression of IFI44 and TRAIL in CD8 cells were all upregulated after three weeks of 15-HETE diet (Fig. 4B). Of note, IFI44 was expressed at a very low level in pulmonary vascular cells both in mouse and human (Suppl. Figure 4).

### Intestinal epithelial cell IFNα4 is crucial for IFI44 expression in CD8 cells

**Fig. 5:**
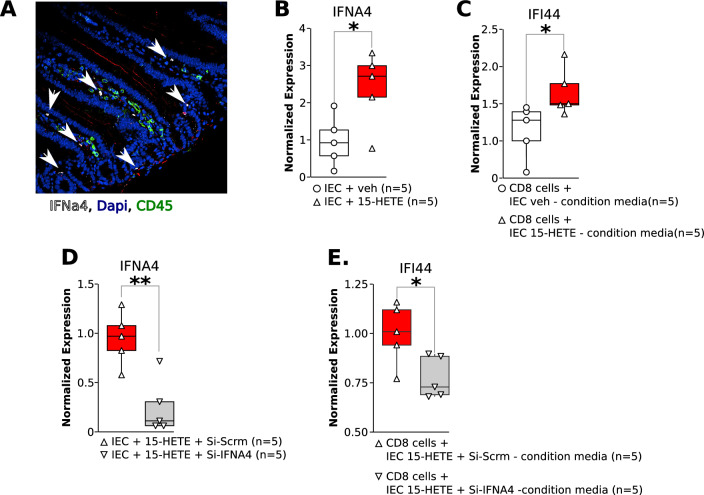
**A** IFNa4 staining on the intestine of 15-HETE diet mice. **B** Quantification of IFNα4 mRNA in intestinal epithelial cells exposed to 15-HETE for 12h. **C** Quantification of IFI44 in CD8 cells exposed to the media of intestinal epithelial cells from panel A for 24h. **D** Quantification of IFNα4 mRNA in intestinal epithelial cells transfected with an Si-Scrm or Si-IFNα4 and exposed 24h later to 15-HETE for 12 h. **E** Quantification of IFI44 in CD8 cells exposed to the media of intestinal epithelial cells from panel C for 24h.

To assess the role of the intestine in producing IFNα4, we found that intestinal epithelial cells express IFNa4 (Fig. 5A). Treating intestinal epithelial cells with 15-HETE was able to significantly up-regulate IFNα4 expression (Fig. 5B). CD8 cells exposed to the conditioned media of intestinal epithelial cells treated with 15-HETE resulted in the upregulation of IFI44. (Fig. 5C). To examine if IFNa4 is solely responsible for IFI44 up-regulation in CD8 cells, we silenced IFNα4 in intestine epithelial cells treated with 15-HETE. Our data shows cultured intestine epithelial cells transfected with Si-IFNα4 before exposing them to 15-HETE (Fig. 5D) had significantly decreased IFNa4 expression. More importantly, expression of IFI44 in CD8 cells exposed to the conditioned media of these epithelial cells significantly reduced (Fig. 5E). Furthermore, CD8 cells exposed to IFNα4 alone also up-regulate IFI44 (Suppl. Figure 5). These data suggest that IFNα4 promotes the expression of IFI44 in CD8 cells in 15-HETE diet-induced PH.

### IFNR-Knockout mice do not develop PH on *15-HETE diet*

**Fig. 6:**
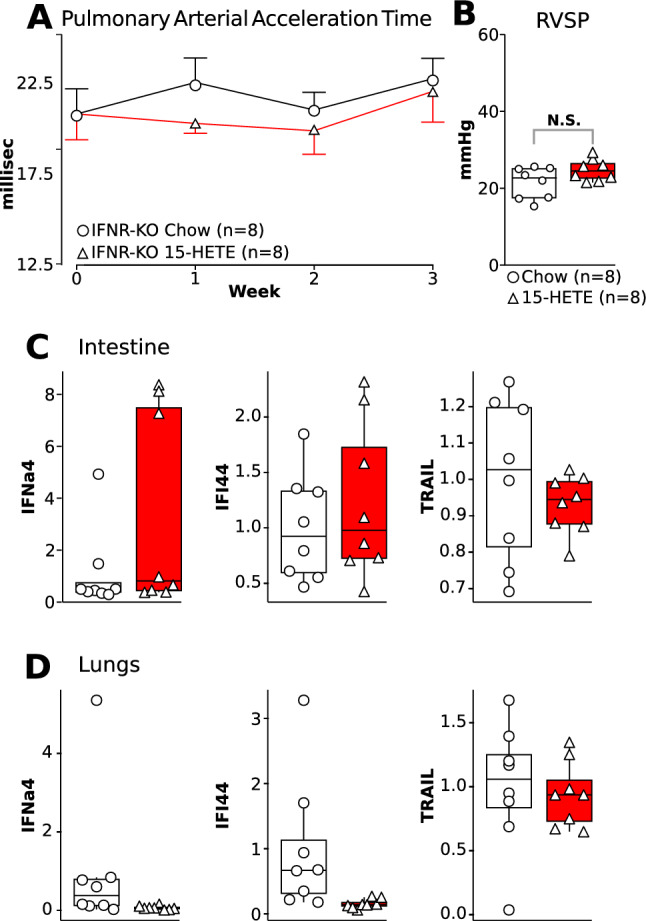
**A** Time-course of pulmonary arterial acceleration time in IFNR-KO mice fed with Chow and 15-HETE diet. **B** Right ventricular systolic pressure (RVSP) at the end of the three-week protocol. **C** mRNA expression of IFNa4, IFI44, and TRAIL in the intestine of IFNR-KO mice fed with Chow and 15-HETE diet. **D** mRNA expression of IFNa4, IFI44, and TRAIL in the lung of IFNR-KO mice fed with Chow and 15-HETE diet.

To examine the role of IFNa4 pathway on 15-HETE-induced PH, we fed IFN receptor Knockout mice with a 15-HETE diet for 3 weeks (Fig. 6). Our data demonstrate that IFNR-KO mice fed with 15-HETE diet do not develop PH as PAAT (Fig. 6A) and RVSP (Fig. 6B) were not significantly different between mice on Chow or 15-HETE diet. In addition, the expression of IFNA4, IFI44, and TRAIL in the intestine and the lungs of IFNR-KO mice were not significantly different between Chow vs 15-HETE diet (Fig. 6C, D). These data support our in vitro findings and demonstrate that IFNA4/IFI44/TRAIL axis is necessary for 15-HETE diet-induced PH.

### Knockdown of IFI44 inhibits 15-HETE-induced PH progression in mice

**Fig. 7:**
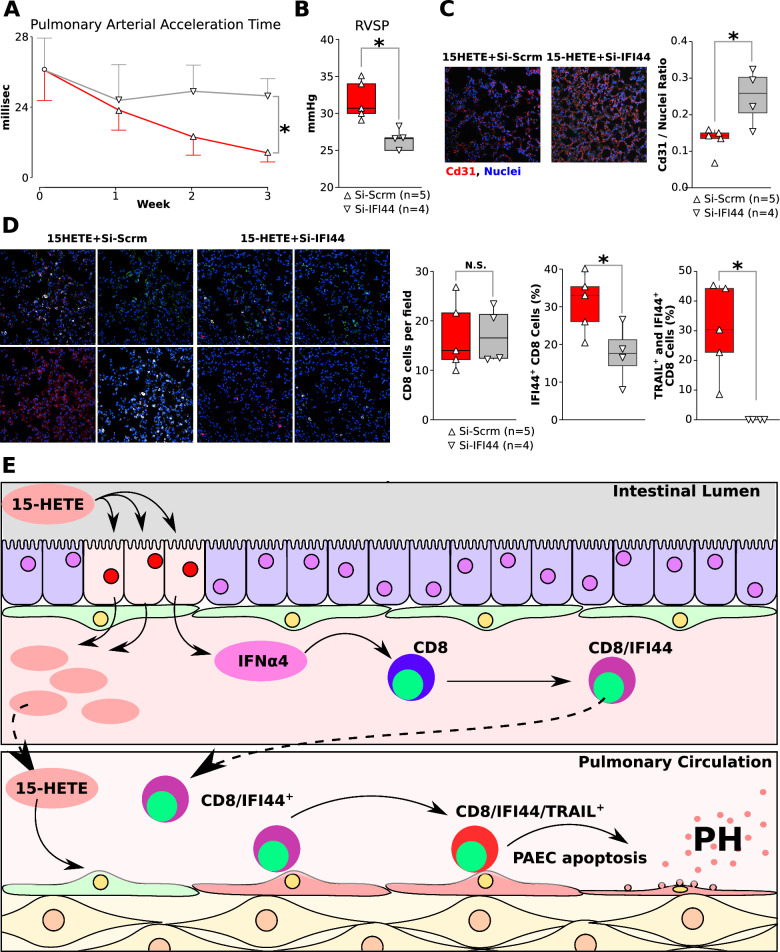
**A** Time-course of pulmonary arterial acceleration time in 15-HETE diet mice treated with either siRNA targeting IFI44 or scrambled siRNA by intratracheal instillation from day 1. **B** Right ventricular systolic pressure (RVSP) in Si-IFI44 and Si-Scrm mice at the end of three week protocol. **C** Representative images and quantification of pulmonary endothelial cell density in Si-IFI44 and Si-Scrm treated 15-HETE diet mice. **D** Representative images and quantification of CD8 cells, CD8 cells expressing IFI44 and CD8 cells expressing IFI44 and TRAIL in Si-IFI44 and Si-Scrm treated 15-HETE diet mice.

We next evaluated the therapeutic potential of IFI44 inhibition in the development of PH by silencing expression of IFI44 in the lungs of mice on 15-HETE diet for 3 weeks from the start of the diet (Fig. 7). Weekly measurements of the pulmonary arterial acceleration time (PAAT) by echocardiography showed in a time dependent decrease of the PAAT in Si-Scrm, while PAAT remains stable in Si-IFI44 treated mice. This difference in PAAT between Si-Scrm and Si-IFI44 treated mice is significant at week 3 of 15-HETE diet (Fig. 7A). Right heart catheterization at the end of the experiment confirmed silencing IFI44 significantly decreased PH severity since *i)* Si-IFI44-treated mice had a significantly lower right ventricular systolic pressure (RVSP) compared to Si-Scrm-treated mice (Fig. 6B); and *ii)* IFI44 silencing led to a significantly higher lung vascular bed density (Fig. 7C). Furthermore, decreased PH severity by silencing IFI44 was associated with significantly lower expression of IFI44 and TRAIL in CD8 cells in the lung (Fig. 7D).

## Discussion

We show that IFI44 is the only gene commonly up-regulated between the intestine of 15-HETE diet mice, and the lungs of 15-HETE diet mice and PAH patients (Fig. 1). We demonstrated that in PBMCs from PAH patients IFI44 is up-regulated and correlates with TRAIL expression (Fig. 2). We also showed that IFNα4, a known inducer of IFI44 is up-regulated in the lungs of PAH patients alongside IFI44 and TRAIL and that IFI44 and TRAIL are expressed by CD8 cells. (Fig. 4). In 15-HETE diet fed mice, we demonstrated that IFNα4 and IFI44 are up-regulated in the intestine as early as one week after starting the 15-HETE diet and from week two they are up-regulated in the lungs of 15-HETE diet mice (Fig. 3). While the number of CD8 cells in the lungs of PAH patients and 15-HETE diet mice is similar to controls, the number of CD8 cells expressing IFI44 and TRAIL is increased (Fig. 4). In vitro, we confirmed that intestinal epithelial cells exposed to 15-HETE upregulate the expression of IFNα4 and that IFI44 expression by CD8 cells is under IFNα4 regulation (Fig. 5). In addition, we show IFNR-KO mice do not develop PH on 15-HETE diet (Fig. 6) further supporting the role of IFNA4/IFI44 in mediating PH by 15-HETE diet. Finally, we show silencing IFI44 in the lungs of 15-HETE diet mice prevents the development of pulmonary hypertension (Fig. 7A–D).

The present research approach converges the power of large-scale transcriptomic analyses with genetically modified murine models and targeted molecular and histological analyses to unravel the complex molecular mechanisms underlying PH development. By integrating human data with the novel 15-HETE diet-induced PH model, we uncovered the intricate role of the intestine in PH, revealing IFI44, as a novel therapeutic target.

The intestine is involved in the development of several cardiovascular and pulmonary diseases including atherosclerosis, heart failure and COPD [11, 29–31]. Since the intestine and the lungs share a common embryonic origin, they also share similarities in structure and immune response [32, 33]. It is now well established that communication between the intestinal tract and the lungs exists via the blood stream and it has been reported that 60% of patients with inflammatory bowel disease had some degree of subclinical lung disease [34].

We have previously shown that 15-HETE diet triggers PH in mice by T-cell-dependent apoptosis of endothelial cells. In patients with PAH, CD8^+^ T cells promote disease development by triggering pulmonary vascular remodeling [35–37]. Indeed, recent studies in PAH patients have found elevated CD8 + cells in the pulmonary vessels [38], in immune cell infiltrate in idiopathic PAH patients [39] and in peripheral blood from PAH patients [40].

We show that the 15-HETE diet induces IFNα4 expression in the intestine in mice in vivo (Fig. 3) and in intestinal epithelial cells in vitro (Fig. 5). We also show that within the intestinal cell conditioned media IFNα4 is solely responsible for inducing IFI44 expression in CD8 + cells (Fig. 5). IFNα4 is part of the type I interferon family. Type I IFNs are cytokines that, among other functions, increase the cytotoxicity of T-cells [41], thus playing essential roles in the inflammatory response. Interestingly, the European Society of Cardiology lists IFNα4 as a potential PAH-inducing toxin in the recommendations for diagnosis and treatment of PAH [42]. Indeed, there are many reports on patients undergoing type 1 IFN therapy that develop PAH [43, 43–45] and that the risk of developing PAH is several-fold higher in patients undergoing IFN therapy than in the general population [46]. Lung cells and blood-derived endothelial cells from PAH patients were found to be more sensitive to the effects of type I IFNs than those from healthy donors [47]. Additionally, protein expression of the IFN alpha/beta receptor (IFNAR1) was significantly increased in the lungs of PAH patients compared to controls. Accordingly, IFNAR1-KO mice subjected to hypoxia had attenuated RV and pulmonary vascular remodeling as compared to controls [47]. Our data further support the role of IFN in PH pathogenesis as IFNR-KO mice are also protected against 15-HETE diet induced PH (Fig. 6). Type I IFNs in the intestine play a crucial role in maintaining homeostasis during inflammation and in response to changing diets [48, 49]. Indeed, the gut microbiome as well as a high-fiber diet were previously shown to protect against pulmonary viral infections by modulating type I IFN responses [50, 50–52]. Previously, it has been demonstrated that diet-derived short chain fatty acids may modulate CD8 + T-cell function during influenza infection and thus modulate airway pathology [53]. To the best of our knowledge, our study is the first to demonstrate the role of 15-HETE diet-induced intestinal IFNα4 in CD8 + T-cell mediated PH induction.

We demonstrate that IFI44 expression is upregulated in PH mouse lungs and PAH human lungs (Fig. 1). We furthermore show upregulated IFI44 in PBMCs from PAH patients and in pulmonary CD8 + cells from human PAH patients compared to controls (Fig. 2). We show for the first time that IFI44 deficiency in the lungs attenuates PH development in mice (Fig. 7). IFI44 has been reported to be upregulated in the lungs of an infant patient suffering from interstitial lung disease [54]. IFI44 was also found to be upregulated in human lung organoids infected with SARS-CoV-2 [55]. Lastly, upregulated IFI44 was observed in mesenchymal progenitor cells of PAH patients as compared to controls [56]. IFI44 has also been implicated as a biomarker in several immune disorders, including lupus [57, 58], Sjögren's syndrome and immune cell infiltration [59] and psoriasis [60]. Whether IFI44 could also serve as a biomarker for PAH as well remains to be elucidated. To that end, interestingly, our study demonstrates that IFI44 is upregulated in the lungs of PAH patients and in mice fed a 15-HETE diet, where IFI44 plays a role in the activation of CD8 T cells. We also show that both in PAH patient lungs, and 15-HETE diet mouse lungs, the absolute number of CD8 + T cells in the lungs are not different from their respective controls. However, it has been reported that in MCT rat model of PH, the number of CD8 + T-cell numbers are reduced both in the lungs and in peripheral blood. While as of yet no reports exist on CD8 + T-cells in the SuHx model, we and others have reported that CD4 + T-cells are downregulated in SuHx rat lungs. Together these reports allude to differential regulation of T cells in different PH animal models. As such, the decreased expression of IFI44 in the lungs of MCT and SuHx rats (Suppl. Figure 2) may be related to overall decreased T cell numbers in the MCT and SuHx models, while CD8 + T cell numbers in the 15-HETE model are not changed.

IFI44 expression in PAH is correlated with expression of death receptor ligand TRAIL (Fig. 2). We show that TRAIL is upregulated in human CD8 cells in PAH lungs (Fig. 2). We furthermore demonstrate that 15-HETE diet enhances TRAIL expression in mouse lungs and in the pulmonary CD8 cells specifically. Moreover, we show that IFNα4 stimulates TRAIL expression in CD8 cells, which is dependent on IFI44 (Fig. 5). TRAIL is mainly expressed on the plasma membrane of immune cells, where it plays a critical role in inducing apoptosis of target cells [61]. As such, TRAIL has been shown to play crucial roles in immune surveillance of tumors, limiting virus infections [62], and protection against development of diabetes [63]. However, TRAIL was also shown to inhibit apoptosis and promote survival in various cancer cell types [61]. As such, the roles of TRAIL seem to be highly context dependent [64, 65]. TRAIL has previously been implicated in PAH pathobiology [65]. Elevated TRAIL expression was previously found in lungs [66], pulmonary vascular lesions [67], and serum [68] of PAH patients. Investigations into the causal role of TRAIL in MCT and Sugen/hypoxia rodent models revealed that TRAIL promotes PH by enhancing PASMC proliferation, migration, and vascular remodeling, as well as stimulating proinflammatory cytokine production in PASMC [66, 69, 69, 70]. Our data show TRAIL expression is decreased in the intestine whereas it increases in the lungs of mice at the end of 3 week 15-HETE diet (Fig. 3). Hence the activation of TRAIL seems to be lung specific, the reason behind this remains to be investigated. In the present study, we add new evidence on the role of TRAIL as not only participating in vascular remodeling but also in the loss of pulmonary vascular bed by functioning as an IFI44 target (Figs. 5, 7).

Here, we assessed the pulmonary vascular bed by employing the CD31-to-nuclei ratio to identify and count pulmonary vessels as a surrogate of pulmonary vascular perfusion, conceptually similar to the capillary-to-alveoli ratio used previously by other investigators [71]. We used this quantification on histological images of lung parenchyma where no muscularized vessels were visible. This method allowed us to reproduce previous findings of decreased vascular perfusion in PAH patients and to demonstrate the loss of pulmonary vascular perfusion in 15-HETE diet mice.

A limitation of our study is that we were not able to elucidate whether the IFI44^+^ and IFI44^+^/TRAIL^+^ CD8 cells observed in PH lungs migrate from the intestine. Investigating the origin of these CD8 cells would require tracking CD8 cells solely activated in the intestine. To our knowledge, a tracer specifically activated in the intestine and not in any other organ is currently not available. Nonetheless, future studies where CD8 cells are tracked over time upon 15-HETE diet initiation will unravel the origins of IFI44^+^ and IFI44 + /TRAIL + CD8 cells in the lungs. While we used intratracheal siRNA instillations to knock down IFI44 in CD8 + cells in the lung, we do not know the effects of IFI44 knockdown in other pulmonary cell types such as smooth muscle cells or endothelial cells on PH development and whether this could also contribute to improved outcome. Another limitation of our study is that a definitive answer on the causal and temporal role of the intestine in context of 15-HETE/IFNα4/IFI44-induced PH will only be unraveled using an intestinal epithelial-specific IFNα4 knockout mouse model. Future studies will further elucidate these cell-specific mechanisms.

Taken together, our data provide new evidence for a crucial role of the intestine and diet in the development of pulmonary hypertension. Our results demonstrate a diet-induced inflammatory response through the intestinal IFNα4-mediated activation of IFI44/TRAIL axis in CD8 cells. This study paves the way to a better understanding of the role of the intestine in the development of pulmonary vascular diseases.

## Methods

### Bioinformatic analysis

The small intestine and lungs of C57Bl6 wildtype mice fed a chow and 15-HETE diet were dissected, and RNA was extracted using mirVana for the intestine and Trizol, followed by a cleanup using RNeasy MinElute Cleanup Kit (Qiagen) according to manufacturer’s instructions, for the lungs. We performed RNA-sequencing on Illumina HiSeq3000 for a single-end 1 × 50 run. The reads were mapped and quantified by STAR 2.7.9a [21] using the mouse genome GRCm39. In Partek Flow (Partek® Flow® software, v7.0 Copyright ©. 2019 Partek Inc., St. Louis, MO, USA.). Read counts were normalized by CPM + 1.0E-4. Statistical analysis comparing chow diet versus 15-HETE diet was performed on the gene count matrix using the DESeq2 R package [22]. Genes were considered differentially expressed for an adjusted p-value below 0.05 and an absolute fold change above 1.5. Mouse lung RNA-sequencing was generated previously [6]. Human lung microarray data were obtained from GSE117261 [18–20]. We compared failed lung donor for transplantation (*n* = 25) to idiopathic pulmonary arterial hypertension samples (*n* = 32). A human PBMC microarray was obtained from GSE33463 [23]. We compared healthy individuals (*n* = 41) versus patients with pulmonary arterial hypertension associated with systemic scleroderma (*n* = 42). In both cases, differential expression analysis was done using the R limma package (R Core Team (2018) [24].

Differentially expressed genes from mouse intestine and lung, and human lung were cross-referenced to identify genes that were similarly dysregulated between all three datasets. For PBMC datasets, all differentially expressed genes were correlated with IFI44 expression (Spearman correlation). All genes with a Spearman coefficient above 0.7 were used to identify the genes that are classified by gene ontology as part of Extracellular Region (GO:0005576), Positive regulation of apoptotic process (GO:0043065), and Signaling Receptor Binding (GO:0005102) [25].

### Human subjects

Human lung samples were obtained from the pulmonary hypertension breakthrough initiative (PHBI). The patient characteristics are summarized in Table 1. Definition of abbreviations: 6MWD = 6-min-walk distance; mPAP = mean pulmonary arterial pressure; PAH = pulmonary arterial hypertension; PDE5 = phosphodiesterase 5; PVR = pulmonary vascular resistance; Values are expressed as mean ± SD.

**Table 1:** Patients Characteristics

Parameters	Ctrl (*n* = 18)	PAH (*n* = 9)
Sex (% female)	44	44
Age (years)	44.4 ± 14.3	42.8 ± 12.1
mPAP (mmHg)	–	59.7 ± 16.7
PVR (Woods Unit)	–	12.5 ± 5.3
6MWD (m)	–	334 ± 96.4
Medication (*n*, (%))	–	
PDE5 inhibitor	–	8 (88)
Endothelin receptor antagonist	–	8 (88)
Prostacyclin analog	–	8 (88)

### In vivo mice experiments

The institutional Animal Research Committee approved all animal procedures (ARC-2010–045) which are according to current NIH guidelines. IFNR1-KO (028288 B6(Cg)-Ifnar1tm1.2Ees/J) male mice were purchased from Jackson Laboratories. Wild-type male, 6–8 week old, C57Bl6/J mice were purchased form Charles River and Jackson Laboratories. To induce PH, mice were fed chow (Teklad 7013) supplemented with 15-HETE (5ug/day, Cayman Chemical, 34,720) for 3 weeks. Regular chow-fed mice served as controls. For time course experiments, some mice on 15-HETE diet were sacrificed at 1-, 2- or 3-weeks after diet initiation. For in-vivo silencing of IFI44, mice on 15-HETE diet received intratracheal instillations of siRNA targeting IFI44 (Horizon, A-051791–13-0050, 5 nmol) or a scrambled control siRNA (Horizon, #D-001910–01-50, 5 nmol) from day 0 every 3–4 days for a total of 6 instillations over 3 weeks. PH severity was monitored weekly by Doppler echocardiography (Vevo 3100, VisualSonics) under 3% isoflurane anesthesia. Pulmonary artery blood flow was measured to assess pulmonary arterial acceleration time (PAAT). At the end of the 3-week diet, right ventricular systolic pressure (RVSP) was measured via open-chested right heart catheterization under 3 isoflurane anesthesia. Mice were euthanized via excision of heart and lungs while still under deep anesthesia.

### Immunofluorescence

Mouse lungs were embedded in OCT and were sectioned at 5 μm. Human lung tissue sections in paraffin were obtained from PHBI. Paraffin or OCT compound were removed from lung tissue sections, after which antigen retrieval was performed by heat induced antigen retrieval in citrate buffer pH 6 for 30 min. Sections were blocked with 5% goat serum for 1 h at room temperature. Antibodies against CD31 (1:200, Novus Biological NB100-2284), CD8 (1:100, ThermoFisher 14–0808-82), IFI44 (1:100, ThermoFisher PA5 96,967), or TRAIL (1:200, Sigma HPA054938) CD45 IFNa4 were incubated overnight at 4 °C. Secondary Alexa Fluor antibodies (1:1000, Invitrogen, A11001, A21245, and A11012) were incubated for 1 h at room temperature. Slides were mounted in Fluoromount G with DAPI (ThermoFisher #00–4959-52) and imaged using a Nikon A1 confocal microscope.

Lung vascular bed density was calculated as a ratio of CD31 staining surface over the total nuclei surface in five random fields per tissue section. IFI44 + /CD8 + and TRAIL + /IFI44 + /CD8 + cells were counted and calculated as the ratio of total CD8 + cells in at least five random fields per tissue section.

### Cell experiments and treatments

Healthy rat small intestine epithelial cells (IEC-6) were purchased from ATCC (CRL-1592) and cultured in DMEM supplemented with 0.1 Unit/ml human insulin, 90%; fetal bovine serum (FBS; ATCC 30-2020), 10%. Intestinal epithelial cells were stimulated with 15-HETE (10uM, Cayman Chemical, 34,720) for 24 h. IFNα4 was silenced in intestinal epithelial cells using siRNA (50 nM, Dharmacon Cat #A-098320–13-0010) or scrambled control (50 nM, Dharmacon cat #D-001910-01-05)) using Lipofectamine RNAiMAX (ThermoFisher) in Optimem medium (ThermoFisher).

CD8 cells were purchased from ALLCELLS (Lot# 3,003,028) and cultured in RPMI-1640 Medium (ATTC, ATTC 30-2001).

For crosstalk experiments, CD8 cells were stimulated with conditioned media from intestinal epithelial cells or with IFNα4 (100 ng, R&D system, 10,259-IF-010) for 24 h.

### RNA isolation, RT and qPCR

Lung and intestines were flash-frozen in liquid nitrogen and crushed into powder. Total RNA was isolated from tissue powder or cells with Trizol or mirVana (Thermo Fisher) according to manufacturer’s instructions. Reverse transcriptions were performed using the High-Capacity cDNA Reverse Transcription kit (Thermo, #4,368,814) with a mix of poly(A) and random primers. The qPCRs were performed using Power Up SYBR Green Master Mix (Thermo, # A25779) on a BioRad CFX Connect PCR detection system. Primers are listed in Table 2.

**Table 2:** Primer sequences

genes	Forward primer	Reverse primer
Hs SPYRD3*	*ATCGTGGACCCTGGAGAGAA*	*AGCCCATGATGTCCCCTTTG*
*Hs IFI44*	*TGATAAAACATGCTGGAGGCA*	*AGGGCTACTCACATGCCAAC*
*Hs CXCL10*	*GGTGTTCTTTTCCTCTTGGGC*	*AACAGCGACCCTTTCTCACT*
*Hs TRAIL*	*GTGATCTTCACAGTGCTCCTG*	*TGCTTCAGCTCGTTGGTAAAGTA*
*Hs IFNα4*	*GATCCCTCTCGTTTTCAACAAACT*	*TGCCTGCACAGGTATACACCAA*
Ms DDX3X*	*TTTCGGGGTAGTGTGAGCTT*	*TATGGCTACACCGCCAATGC*
*Ms IFI44*	*TCACTTTTGTCTCCCTCACCC*	*AGTCCATTCCCAGTCCTTTCAG*
*Ms TRAIL*	*GCCAGCTCTGCTGTTTTGAG*	*CACCTGGTGGCACTAATGGT*
*Ms IFNα4*	*TGATGGTTCTGGTGGTGATG*	*GAGTGTGAAGGCTCTCTTGTT*
*Rt IFNα4*	*TGATGGTTCTGGTGGTGATG*	*GAGTGTGAAGGCTCTCTTGTT*
Rt PPIA*	*AGCATACAGGTCCTGGCATC*	*TTCACCTTCCCAAAGACCAC*

Housekeeping genes are marked with *. *Hs* Human, *Ms* Mouse, *Rt* Rat

### Statistics

Homogeneity of variance and normality was assessed using Levene’s test and Shapiro–wilk’s test respectively. To compare two groups, if variances were equal and the values were normally distributed, we performed a *t*-test. If the variances were equal but the values were not normally distributed we performed a Wilcoxon test. In case the variances were not equal and the values were normally distributed we performed a Welch *t*-test. When the variances were not equal and the values were not normally distributed, values were log transformed and t-test was performed on the log transformed data.

To compare more than two independent groups, when the variances were equal and the values were normally distributed, we performed a one-way ANOVA followed by a Tukey multiple comparison test if the overall ANOVA was significant. If the variances were equal and the values were not normally distributed, we performed a Kruskal–Wallis test followed by a Wilcoxon multiple comparison test if the overall Kruskal–Wallis test was significant. If the variances were not equal and the values were either normally distributed or not, values were log transformed and one-way ANOVA and Tukey multiple comparison tests were performed on the log transformed data, if the overall one-way ANOVA was significant.

To perform a statistical test on multiple groups with repeated measures, we performed a two-way ANOVA followed by Tukey multiple comparison test if the overall ANOVA was significant. If the variances were equal and the values were normally distributed, statistical tests were performed on the original value. If the variance were equal and the values were not normally distributed, the values were log transformed and two-way ANOVA and Tukey multiple comparison tests were performed on the log transformed data. Values were also log transformed if the variances were not equal whether the values were normally distributed or not.

To assess the strength and magnitude of associations between continuous measures, we used Pearson’s correlation coefficient. A significance level of 5% (p < 0.05) was considered statistically significant.

## Supplementary Information

Additional file1 (DOCX 939 KB)

## Data Availability

The data, analytic methods, and study materials will be made available to other researchers on reasonable request for purposes of reproducing the results or replicating the procedure. Online transcriptomic data are identified within the manuscripts.
