# Supplementary material for: Intestinal IFNα4 promotes 15-HETE diet-induced pulmonary hypertension
Source: Respir Res. 2024 Nov 28;25:419. doi: 10.1186/s12931-024-03046-z (PMC11606228; doi:10.1186/s12931-024-03046-z)
Supplement: Supplementary file 1 — Additional file1 (DOCX 939 KB) [file 12931_2024_3046_MOESM1_ESM.docx]

**A novel gut-lung axis in 15-HETE diet-induced pulmonary hypertension**

**Supplemental Figures**

*Grégoire Ruffenach, Ph.D.^1^; Lejla Medzikovic, Ph.D.^1^; Laila Aryan, PhD^1;^ Wasila Sun BS^1^; Long Lertpanit, MS^2^, Ellen O’Connor, Ph.D.^2^, Ateyeh Dehghanitafti, M.D.^1^, Mohammad Reza Hatamnejad, MD, MPH^1^, Min Li, Ph.D.^1^; Srinivasa T. Reddy, Ph.D.^2^; *Mansoureh Eghbali, Ph.D.^1^

^1^Division of Molecular Medicine, Department of Anesthesiology and Perioperative Medicine, David Geffen School of Medicine, University of California, Los Angeles. ^2^ Division of Cardiology, Department of Medicine, David Geffen School of Medicine, University of California, Los Angeles.

*Corresponding author: Grégoire Ruffenach, Ph.D. ([gr.ruffenach@laposte.net](mailto:gr.ruffenach@laposte.net)) and Mansoureh Eghbali, PhD. ([meghbali@ucla.edu](mailto:meghbali@ucla.edu)). Department of Anesthesiology, Division of Molecular Medicine, David Geffen School of Medicine at University of California Los Angeles, CHS BH-550 CHS, Los Angeles, CA, 90095-7115, USA

Country affiliation : United States of America

Running title: Gut-lung axis in PH.

Conflict of Interest: None

Funding: This work was supported by the American Heart Association 20POST35210727 (G.R.), 20CDA35350059 (G.R.), and 23POST1022457 (L.M), the UCLA and Caltech integrated Cardiovascular Medicine for Bioengineers T32HL144449 (EO) and the National Institutes of Health R01HL162124 (M.E and S.T.R), R01HL129051 (M.E. and S.T.R), and R01HL159865 (M.E.).

Running title: Gut-lung axis in PH.

**
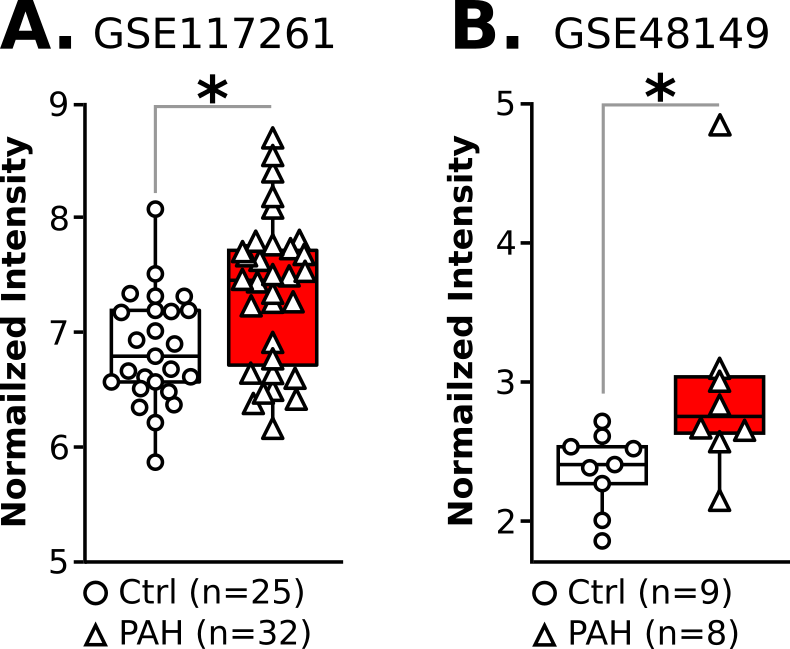
**

**Supp. Fig. 1:** Expression of IFI44 in other PAH patients’ lungs large scale transcriptomic datasets. In two other independent cohort of PAH patients - (**A**) and (**B**) - IFI44 is significantly up-regulated.


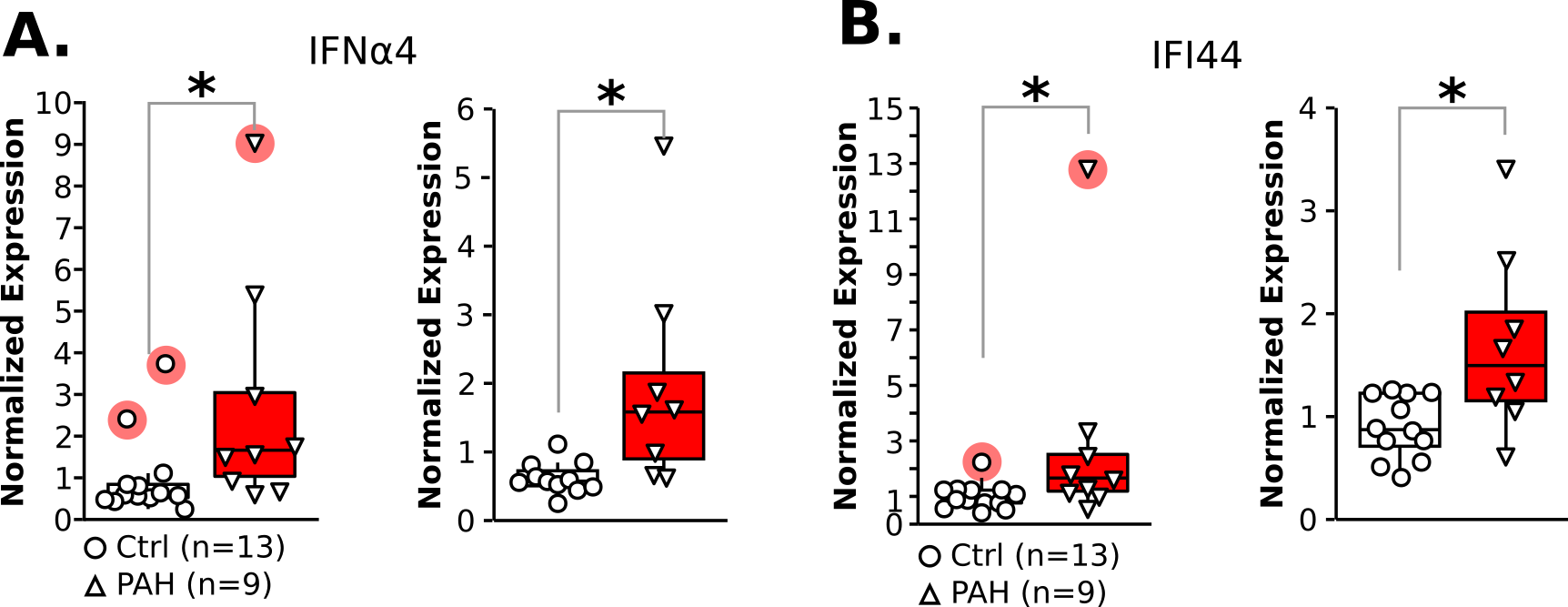
**Suppl. Fig .2**: Outlier detection in Human PAH lung PCR. For the quantification of IFNa4 and IFI44 in the lung of PAH patients, outliers were detected (value above 2 time SD, highlighted in red circles). The statistic was done with and without the outlier to confirm that they are not driving the increase of these gene in our samples. We found that even without these statistical outliers the up-regulation of IFNa4 and IFI44 remain significant. We choose to present the data with the outliers as they represent the biological dispersion of these genes in our PAH patients cohort.


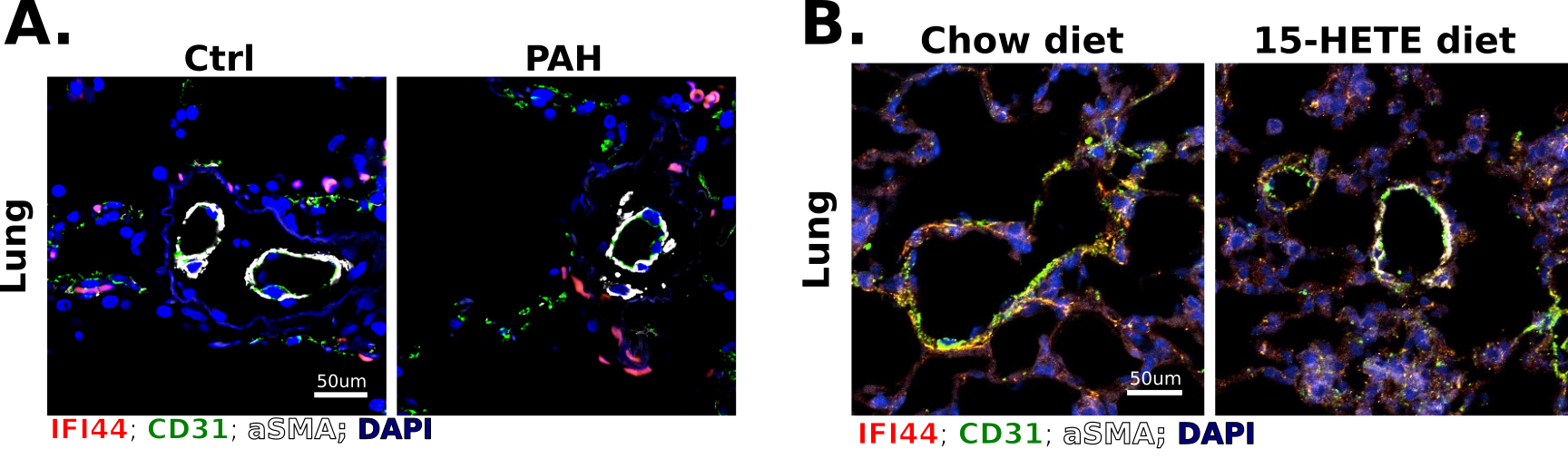


**Suppl. Fig. 3:** IFI44 staining in vascular cells in (**A**) human lungs and (**B**) mice lungs showing no difference in IFI44 expression between Ctrl and PAH or Chow diet and 15HETE diet in vascular cells.


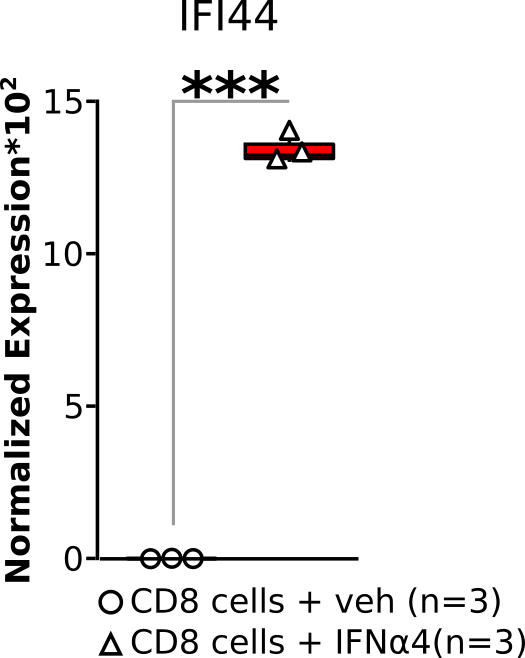


**Suppl Fig 4:** CD8 Cells exposed to IFNa4 up-regulate the expression of IFI44.


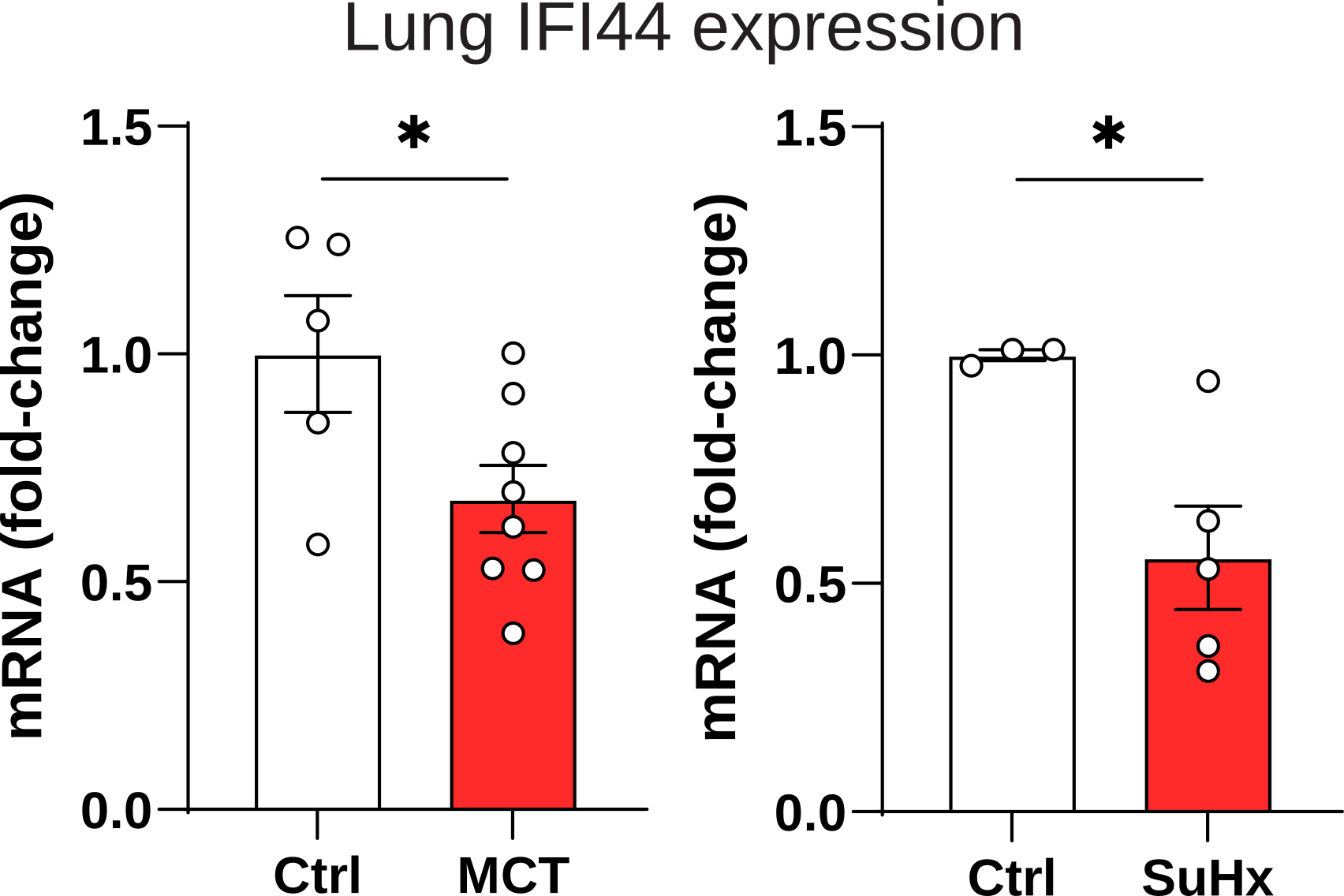
**Suppl Fig 5 :** IFI44 Expression in Monocrotaline (MCT) and Sugen Hypoxia (SuHx) rat models of Pulmonary hypertension. Samples are from previous studies^72, 73^
